# Effort-based decision making in schizotypy and its relationship with amotivation and psychosocial functioning

**DOI:** 10.3389/fpsyt.2023.1123046

**Published:** 2023-02-16

**Authors:** Ryan Sai Ting Chu, Co Co Ho Yi Tong, Corine Sau Man Wong, Wing Chung Chang, Wesley Chor Yin Tang, Charlotte Cheuk Lok Chan, Simon S. Y. Lui, Lai Ming Hui, Yi Nam Suen, Kit Wa Chan, Ho Ming Lee, Eric Yu Hai Chen

**Affiliations:** ^1^Department of Psychiatry, LKS Faculty of Medicine, School of Clinical Medicine, The University of Hong Kong, Hong Kong, Hong Kong SAR, China; ^2^LKS Faculty of Medicine, School of Public Health, The University of Hong Kong, Hong Kong, Hong Kong SAR, China; ^3^State Key Laboratory of Brain and Cognitive Sciences, The University of Hong Kong, Hong Kong, Hong Kong SAR, China

**Keywords:** Effort-based decision-making, effort allocation, schizotypy, amotivation, psychosocial functioning

## Abstract

**Introduction:**

Suboptimal effort-based decision-making with reduced willingness to expend effort for high-probability/high-value reward is observed in schizophrenia patients and is related to diminished motivation, but is understudied in schizotypy. This study aimed to examine effort-allocation in schizotypy individuals and its association with amotivation and psychosocial functioning.

**Methods:**

We recruited 40 schizotypy individuals and 40 demographically-matched healthy controls, based on Schizotypal Personality Questionnaire-Brief (SPQ-B) score (top and bottom 10% SPQ-B scores, respectively), from 2400 young people aged 15-24 years participating a population-based mental health survey in Hong Kong and examined effort-allocation using the Effort Expenditure for Reward Task (EEfRT). Negative / amotivation symptoms and psychosocial functioning were assessed by the Brief Negative Symptom Scale (BNSS) and the Social Functioning and Occupational Assessment Scale (SOFAS), respectively. Schizotypy individuals were categorized into high-amotivation and low-amotivation groups based on a median-split of BNSS amotivation domain score.

**Results:**

Our results showed no main group effect (in either two or three-group comparison) on effort task performance. Three-group comparison analyses on selected EEfRT performance indices revealed that high-amotivation schizotypy individuals displayed significantly less increase in effortful options from low-value to high-value reward (reward-difference score) and from low-probability/low-value to high-probability/high-value reward (probability/reward-difference score) than low-amotivation individuals and controls. Correlation analyses demonstrated trend-wise significance between BNSS amotivation domain score and several EEfRT performance indices in schizotypy group. Schizotypy individuals with poorer psychosocial functioning tended to exhibit smaller probability/reward-difference score relative to other two groups.

**Discussion:**

Our findings indicate subtle effort-allocation abnormalities in schizotypy individuals with high levels of diminished motivation, and suggest the link between laboratory-based effort-cost measures and real-world functional outcome.

## Introduction

Schizotypy refers to a broad phenotype of schizophrenia-like psychopathology, and has been considered as the latent personality organization associated with liability for the development of schizophrenia (1–3). More recently, a full dimensional model has been applied in conceptualizing schizotypy which is regarded as a constellation of stable traits linking a continuum of subclinical and clinical manifestations of schizophrenia-spectrum disorders and normal personality variation expressed in the general population (4, 5). Literature has found that schizotypal traits are associated with cognitive impairment, neuroanatomical abnormalities and genetic risk factors that are qualitatively similar, albeit attenuated, to patients with schizophrenia (6–9). Examining schizotypy in a non-clinical sample may therefore facilitate elucidation of the etiology as well as identification of potential risk and protective factors of schizophrenia (10), without the confounding effect of medication treatment.

Diminished motivation (or termed amotivation) is a core subdomain of negative symptom construct (11, 12) and a major determinant of functional impairment in schizophrenia (13–15). A large body of research has recently been conducted to investigate neurobiological mechanisms, such as reward processing (16, 17), underlying amotivation in schizophrenia. In particular, effort-based decision-making has been increasingly examined in patients with schizophrenia (18). Accumulating evidence indicates that patients in chronic schizophrenia and first-episode psychosis displayed suboptimal effort allocation in pursuit of reward, with significantly reduced willingness to select high-effort/high-reward options than healthy controls (19–22). Such reduced effort expenditure was found to be associated with higher levels of amotivation in many (21–28), though not all (29–32) previous studies. Additionally, several past studies have demonstrated significant relationship between abnormal effort allocation and poor psychosocial functioning (21, 24, 27, 33). It is recognized that individuals with schizotypal traits may experience attenuated negative symptoms including reduced motivation and social drive, which are related to poorer psychosocial functioning and quality of life (34, 35). Of note, there is a paucity of research assessing the relationship between effort-based decision-making and schizotypy, and mixed findings were observed (36, 37). One previous study showed no difference between high-schizotypy individuals and controls in effort allocation for reward (36), while another report revealed that individuals with elevated social anhedonia expended more effort than controls when the probability of reward was most uncertain (i.e., 50% probability) (37).

In the current study, we sought to investigate effort-based decision-making in Chinese individual with high-schizotypy (schizotypy group) versus low-schizotypy (controls), using the Effort-Expenditure for Reward Task (EEfRT) (38) which is a computerized button-pressing task assessing physical effort allocation in response to varying levels of reward magnitude and probability. Specifically, we aimed to examine the association of effort allocation performance with amotivation severity and psychosocial functioning in the schizotypy group. EEfRT has been well-studied in healthy populations (38, 39) and is the most frequently used performance-based paradigm in studying effort allocation in schizophrenia (20). Furthermore, EEfRT has demonstrated good psychometric properties, tolerability and group discriminability (33, 40). Based on prior studies (20), we hypothesized that individuals in schizotypy group would exhibit suboptimal effort allocation with reduced willingness to exert effort for high-value/high-probability reward compared with controls, and such altered performance would be associated with amotivation. Moreover, we predicted that diminished effort expenditure for reward would be related to poorer psychosocial functioning.

## Methods

### Participants and study setting

Participants in this study were recruited from the Hong Kong Youth Epidemiology Study of Mental Health (HKYES), which is an ongoing territory-wide population-based survey examining mental health conditions in Chinese young people aged 15–24 years in Hong Kong (41). The study adopts a multistage cluster sampling design with stratification by geographical location. Invitation letters were mailed to randomly select household addresses obtained from the local government, and a total of 2,400 participants were recruited between May 2019 and May 2021. During the baseline assessment, participants’ sociodemographic characteristics, severity of various psychiatric symptom domains, presence of major psychiatric disorders, psychosocial variables and functional status were evaluated by trained research assistants *via* structured face-to-face interview (41). In the current study, we applied an extreme-group design and classified individuals with schizotypy based on their scores on the Schizotypal Personality Questionnaire-Brief (SPQ-B) (42, 43). Individuals who had a total SPQ-B score within the top 10% of the HKYES sample were categorized as individuals with schizotypy, whereas those with a total SPQ-B score at the bottom 10th percentile were categorized as healthy controls. Forty individuals with schizotypy were randomly selected from the schizotypy sample and constituted the schizotypy group (mean = 16.7; SD = 1.9) of the current study. Forty demographically-matched controls were then selected from the control sample (mean = 0.3; SD = 0.9) for comparison. All study participants were right-handed. Individuals with lifetime diagnosis or family history of psychotic disorder, past or current use of antipsychotic medications, intellectual disability, a history of substance use disorder, head trauma or neurological disease were excluded from the study. This study was approved by the local institutional review boards and all participants provided written informed consent. For those aged under 18 years, parental consent was also obtained.

### Symptoms, functional, and cognitive assessments

Negative symptoms including amotivation were assessed using the Brief Negative Symptom Scale (BNSS) (44). Following the method of previous research (22, 45), amotivation consisted of items of anhedonia, avolition, and asociality subscales on the BNSS. The Snaith-Hamilton Pleasure Scale–Chinese version (C-SHAPS) (46) was employed as a self-reported measure of anhedonia. Psychosocial functioning was assessed by the Social and Occupational Functioning Assessment Scale (SOFAS) (47). A brief battery of cognitive assessments comprising logical memory subtest from the Wechsler Memory Scale-Revised (WMS-R) (48), digit symbol subscale from the Wechsler Adult Intelligence Scale-Revised (WAIS-R) (48), letter-number span (49), trail making test (50) and letter cancelation test (51) were administered. Additionally, participants completed three 10-s trials of measuring finger-tapping speed using their non-dominant pinky finger prior to EEfRT administration. The average number of presses over three trials was used as a quantitative measure of motor function to evaluate potential group differences in motor speed as a confound.

### Effort-based decision-making task

The Effort-Expenditure for Reward Task (EEfRT) (38) is a computerized, multi-trial button-pressing experiment that assesses a participant’s willingness to expend physical effort for monetary reward. On each trial, participants were asked to choose between a “high-effort” (hard) and “low-effort” (easy) task. Low-effort trials required participants to make 30 button presses within 7 s using their dominant index finger, and hard trials required participants to make 100 button presses within 21 s using their non-dominant pinky finger. The reward value for the easy choice was fixed at $1, while that of the hard choice ranged from $1.24 to $4.12. Receipt of reward following successful completion of a trial was contingent upon three probability levels (12, 50, or 88%), regardless of hard or easy choice. All participants received trials presented in the same pre-randomized sequence, within which there was an equal number of three probability-level trials associated with each level of reward value. Information regarding the probability of receiving reward and the reward value associated with high-effort choice was provided at the beginning of each trial. Following trial completion, participants were provided with feedback about whether the trial was successfully completed, and if so, whether they had won monetary reward for that trial. Task duration was modified to last for 15 min to minimize potential fatigue effect on task performance. Participants were provided with standardized task instructions and underwent four practice trials under the supervision of a trained research assistant. Participants were told that they would receive a bonus at the end of the study based on their task performance, in addition to the base-compensation of HK$100 (US$13) for their participation. In actuality, all participants were compensated HK$200 (US$26).

### Statistical analysis

First, to examine the effects of group, probability and reward magnitude on effort allocation, we performed a 2 group (schizotypy vs. controls) × 3 probability (12, 50, and 88%) × 3 reward (values for hard option were binned into: small = $1.24 to $2.00, medium = $2.01 to $3.00, and large = $3.01 to $4.12) mixed analysis of variance (ANOVA), with proportion of hard choices as the dependent variable. Significant effects were further explored using a series of post-hoc *t*-tests. Second, we derived a number of recommended EEfRT performance indices with reference to previous research (27, 30, 33) between-group comparison and correlation analyses: (1) percentage of hard choices in 88% probability condition; (2) percentage of hard choices at large reward condition; (3) percentage of hard choices in 88% probability with large reward condition; (4) difference in percentage of hard choices between 12 and 88% probability conditions (probability difference score); (5) difference in percentage of hard choices between small and large reward conditions (reward difference score); and (6) difference in percentage of hard choices between 12% probability with small reward and 88% probability with large reward conditions (probability/reward difference score). Third, to investigate the relationships between effort expenditure and negative symptoms, two approaches were adopted in the current study. As evidence indicates that amotivation is specifically linked to impaired effort allocation, we employed a categorical approach by dichotomizing individuals with schizotypy into high (HIGH-AMO) and low (LOW-AMO) amotivation subgroups, based on a median split on BNSS amotivation score (split score = 10.5), for comparison analyses on EEfRT performance (21, 22, 28). We also examined correlations of BNSS total and amotivation scores with various EEfRT performance indices. It is recommended to evaluate the effects of negative and amotivation symptoms both categorically and dimensionally because the construct is not purely continuous, but rather of a hybrid categorical-dimensional nature (52). As we did not observe any participant who only selected hard trials across all reward levels (i.e., all-hard inflexible responder) in either schizotypy or control groups, the entire study sample was included for difference-score analyses (33). Fourth, we conducted correlation analyses to examine the relationship between psychosocial functioning (measured by SOFAS) and EEfRT performance. Additionally, we assessed correlations of EEfRT performance with self-reported anhedonia and cognitive functions. We applied Benjamini–Hochberg procedure with false discovery rate being set at standard 25% for the correlations that were hypothesized-driven (i.e., EEfRT measures with overall negative symptom severity, amotivation levels, and psychosocial functioning), and generated critical values (based on the ranking of *p* values) for each of the variables with *p* < 0.05 to confirm their statistical significance. Bonferroni correction (i.e., more conservative approach) was employed in the case of correlations that were not hypothesis-driven.

## Results

### Characteristics of the sample

Demographics, symptom severity, psychosocial functioning, and cognitive performance of the study participants are summarized in Table 1. Schizotypy and control groups did not differ in age, gender and years of education. There was no group difference in any of the cognitive functions and finger-tapping speed. As expected, individuals with schizotypy had higher levels of negative symptoms and amotivation, and poorer psychosocial functioning than controls. Self-reported anhedonia levels did not differ between the two groups.

**Table 1:** Demographics, symptoms severity, functioning and cognitive performances of schizotypy and control groups.

Variables of Interest^a^	Schizotypy (*N* = 40)	Controls (*N* = 40)	*χ*^2^/*t*	*p*	High-AMO (*N* = 20)	Low-AMO (*N* = 20)	*F*/*χ*^2^	*p*
Demographics								
Age in years	20.83 (2.81)	21.03 (3.15)	−0.30	0.765	21.50 (2.54)	20.15 (2.96)	1.08	0.344
Female gender, *N* (%)	19 (48%)	22 (55%)	0.45	0.502	9 (45%)	10 (50%)	0.55	0.759
Years of education	13.88 (1.77)	14.05 (1.89)	−0.43	0.671	14.35 (1.57)	13.40 (1.88)	1.47	0.237
Symptoms severity and functioning								
BNSS total	15.48 (9.27)	6.73 (7.16)	4.72	<0.001	20.00 (8.52)	10.95 (7.80)	19.96	<0.001^c^
BNSS-AMO^b^	10.30 (6.40)	4.03 (4.25)	5.17	<0.001	15.10 (5.20)	5.50 (2.91)	47.69	<0.001^d^
C-SHAPS	24.33 (6.43)	21.65 (5.82)	1.95	0.055	24.95 (8.17)	23.70 (4.16)	2.09	0.130
SOFAS	68.15 (11.54)	75.25 (8.58)	−3.12	0.003	63.25 (7.98)	73.05 (12.61)	10.67	<0.001^e^
Cognitive performance								
Logical memory	12.38 (4.23)	12.79 (3.31)	−0.48	0.632	12.22 (4.40)	12.53 (4.18)	0.14	0.867
Digit symbol	13.15 (3.68)	14.48 (2.99)	−1.77	0.081	13.15 (4.30)	13.15 (3.05)	1.55	0.220
Letter number span	14.83 (2.93)	14.95 (3.43)	−0.18	0.861	15.05 (1.88)	14.60 (3.73)	0.11	0.893
Trail making A	27.68 (10.69)	25.10 (8.29)	1.21	0.230	26.11 (9.41)	29.25 (11.87)	1.27	0.286
Trail making B	55.20 (22.88)	48.73 (18.80)	1.38	0.171	51.03 (20.26)	59.37 (25.05)	1.76	0.179
Letter cancelation	101.88 (3.24)	101.75 (3.67)	0.16	0.872	102.00 (3.28)	101.75 (3.27)	0.04	0.962
Finger tapping (taps/s)	6.01 (0.83)	6.41 (0.99)	−1.95	0.055	5.94 (0.72)	6.08 (0.94)	1.99	0.143

AMO, Amotivation; BNSS, Brief Negative Symptom Scale; C-SHAPS, Snaith-Hamilton Pleasure Scale-Chinese version; SOFAS, Social and Occupational. Functioning Assessment Scale. ^a^Data of all variables are presented in mean and SD except gender (number and percentages). ^b^BNSS-AMO score was derived according to the method adopted in Strauss et al. (12) and Chang et al. (22). ^c^Post-hoc contrast tests showed that High-AMO group had significantly higher BNSS total score than Low-AMO (*p* = 0.001) and control (*p* < 0.001) groups, while the latter two groups did not differ from each other. ^d^Post-hoc contrast tests showed that High AMO group had significantly higher BNSS-AMO score than Low-AMO (*p* < 0.001) and control (*p* < 0.001) groups, while the latter two groups did not differ from each other. ^e^Post-hoc contrast tests showed that High-AMO group had significantly higher SOFAS score than Low-AMO (*p* = 0.005) and control (*p* < 0.001) groups, while the latter two groups did not differ from each other.

### Effort expenditure for reward task performance in schizotypy and control groups

**Figure 1:**
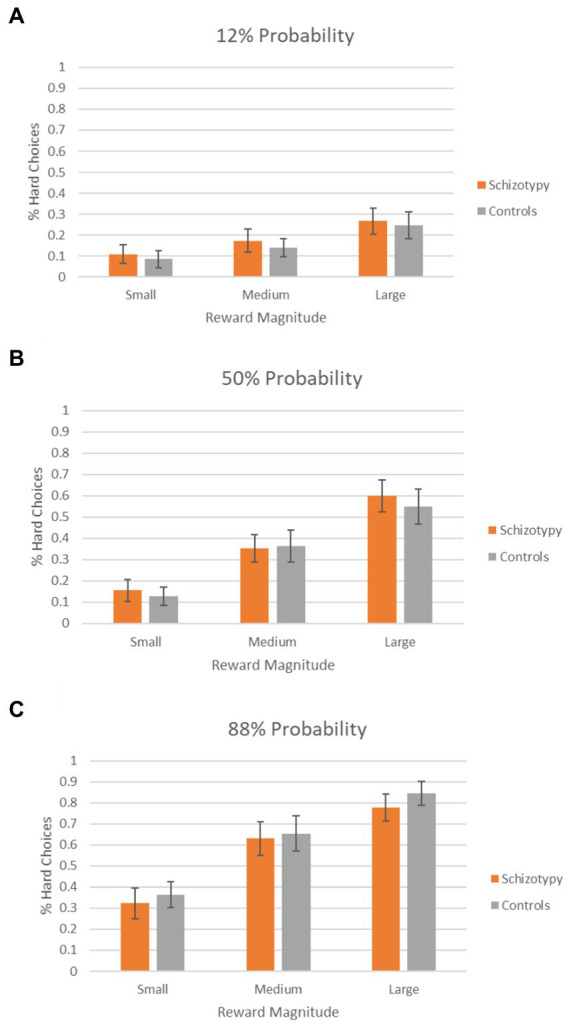
Percentage of hard choices selected by schizotypy group and controls as a function of reward magnitude. **(A–C)** Percentage of hard choices in 12, 50, and 88% probability conditions, respectively.

There were no significant differences between schizotypy and control groups in terms of the number of trials attempted, the number of completed trials, the proportion of hard choices selected and the reaction time (Table 2). The percentage of hard choices for the 2 groups for each task condition is presented in Figure 1. A mixed ANOVA revealed significant main effects of reward magnitude (*F*_1.62,126.19_ = 135.00, *p* < 0.001) and probability (*F*_1.76,137.28_ = 135.03, *p* < 0.001), and a significant reward × probability interaction (*F*_3.62,282.01_ = 19.25, *p* < 0.001). There was no significant main effect of group (*F*_1,78_ = 0.01, *p* = 0.983), interaction effect of reward × group (*F*_1.62,126.19_ = 0.05, *p* = 0.988), probability × group (*F*_1.76,137.28_ = 1.14, *p* = 0.319) or reward × probability × group (*F*_3.62,282.01_ = 0.02, *p* = 0.732). Independent *t*-tests showed no significant group differences in any of the EEfRT performance indices (Table 2).

**Table 2:** EEfRT task performances of schizotypy and control groups.

Variables of Interest	Schizotypy (*N* = 40)	Controls (*N* = 40)	*χ*^2^/*t*	*p*	High-AMO (*N* = 20)	Low-AMO (*N* = 20)	*χ*^2^/*F*	*p*
Basic EEfRT performance								
Trials	63.95 (10.20)	63.80 (9.47)	0.02	0.982	64.45 (9.84)	63.25 (10.78)	0.07	0.929
Hard trial (%)	40.69 (20.56)	40.59 (20.57)	0.02	0.984	38.27 (22.17)	43.10 (19.08)	0.27	0.761
Reaction time	1.27 (0.41)	1.43 (0.44)	−1.65	0.103	1.18 (0.37)	1.37 (0.43)	2.35	0.102
Completed trial	62.00 (10.35)	61.58 (9.61)	0.19	0.850	61.60 (9.56)	62.40 (11.32)	0.50	0.952
Selected EEfRT performance indices								
88% probability	0.61 (0.26)	0.66 (0.26)	−0.84	0.401	0.57 (0.27)	0.65 (0.25)	0.91	0.405
Large reward	0.55 (0.25)	0.56 (0.26)	−0.20	0.984	0.49 (0.27)	0.62 (0.23)	1.51	0.227
88% probability with large reward	0.78 (0.30)	0.85 (0.27)	−1.08	0.283	0.68 (0.32)	0.87 (0.25)	2.88	0.062
Probability difference score	0.42 (0.28)	0.49 (0.27)	−1.20	0.233	0.34 (0.30)	0.49 (0.24)	2.29	0.108
Reward difference score	0.36 (0.25)	0.36 (0.21)	−0.04	0.972	0.26 (0.24)	0.45 (0.23)	3.29	0.042^a^
Probability/reward difference score	0.67 (0.39)	0.76 (0.32)	−1.16	0.249	0.53 (0.43)	0.81 (0.29)	4.09	0.020^b^

AMO, Amotivation; EEfRT, Effort Expenditure for Reward Task. ^a^Post-hoc contrast tests showed that High-AMO group had significantly lower reward difference score than Low-AMO group (*p* = 0.032) only. ^b^Post-hoc contrast tests showed that High-AMO group had significantly lower probability/reward difference score than Low-AMO (*p* = 0.040) and control. (*p* = 0.030) groups. The latter two groups did not significantly differ from each other on probability/reward difference score.

### Effort expenditure for reward task performance in high- and low-amotivation schizotypy subgroups

As shown in Table 1, there were no significant differences in demographics, self-reported anhedonia and cognitive performance between High-AMO schizotypy, Low-AMO schizotypy and control groups. As expected, High-AMO group had significantly higher BNSS total and BNSS-AMO scores than Low-AMO and control groups. High-AMO group also displayed significantly higher SOFAS score than the two groups. Otherwise, the three groups did not differ from each other in the number of trials attempted, the number of completed trials, the proportion of hard choice selected, and the reaction time (Table 2).

**Figure 2:**
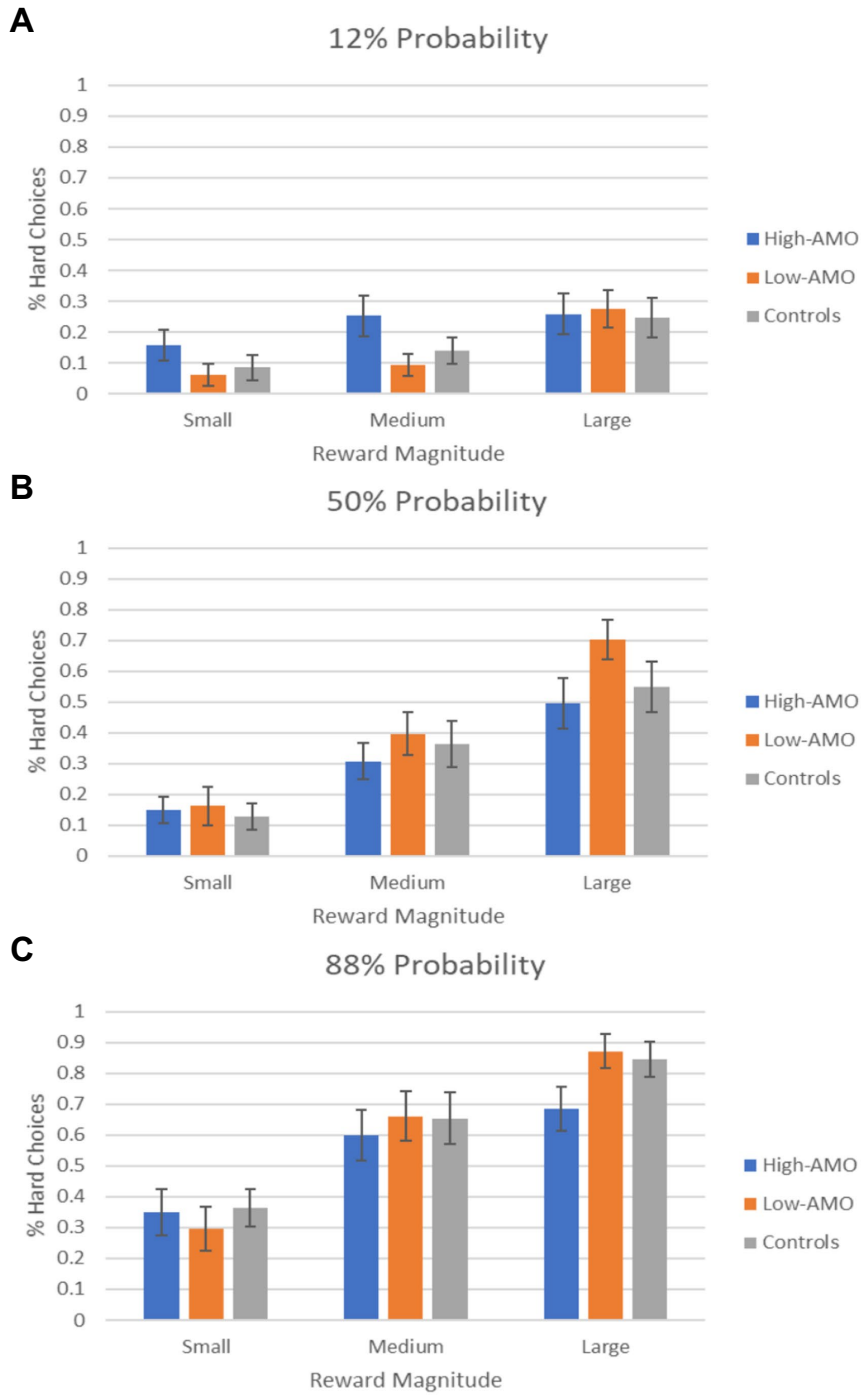
Percentage of hard choices selected by high (HIGH-AMO) and low (LOW-AMO) amotivation schizotypy groups and controls as a function of reward magnitude. **(A–C)** Percentage of hard choices in 12, 50, and 88% probability conditions, respectively.

The percentage of hard choices for each group for each task condition is presented in Figure 2. A 3 × 3 × 3 mixed ANOVA demonstrated significant main effects of reward (*F*_1.62,124.92_ = 127.76, *p* < 0.001) and probability (*F*_1.75,134.41_ = 118.48, *p* < 0.001), significant reward × probability (*F*_3.60,277.42_ = 17.51, *p* < 0.001) and reward × group (*F*_3.25, 124.92_ = 2.62, *p* = 0.049) interactions. There was no significant main effect of group (*F*_2,77_ = 0.11, *p* = 0.897), interaction effect of probability × group (*F*_3.49,134.41_ = 2.34, *p* = 0.070) or reward × probability × group (*F*_7.21,277.42_ = 0.59, *p* = 0.766). Post-hoc contrast tests on reward × group interaction were all non-significant (all *p* > 0.05). Three-group comparisons on EEfRT performance indices found significant difference in reward difference score (*F*_2,77_ = 3.29, *p* = 0.042) and probability/reward difference score (*F*_2,77_ = 4.09, *p* = 0.020) (Table 2). Post-hoc contrast tests demonstrated that High-AMO group had significantly lower reward difference scores than Low-AMO group (*p* = 0.032), and lower probability/reward difference scores than both Low-AMO group (*p* = 0.040) and controls (*p* = 0.030).

### Correlations of effort expenditure for reward task measures with amotivation, psychosocial functioning, and cognition

As shown in Table 3, EEfRT performance indices were not significantly correlated with negative symptoms in individuals with schizotypy. Nonetheless, several correlations between negative symptoms and EEfRT performance indices approached significance, including BNSS-AMO score and reward difference score (*p* = 0.062), BNSS-AMO score and probability/reward difference score (*p* = 0.070), and BNSS total score and reward difference score (*p* = 0.053). Additionally, BNSS total was inversely correlated with the proportion of hard choice in the 88% probability with large reward condition when the analysis was conducted for the entire study sample (also including low-schizotypy, i.e., control group), albeit marginally failed to survive after Benjamini–Hochberg correction (*p* = 0.029 > critical value of 0.021; Supplementary Table S1). Concerning the relationship between EEfRT measures and psychosocial functioning, correlation between SOFAS and probability/reward difference score approached statistical significance (*p* = 0.058) in schizotypy group (Table 3). In the entire sample, SOFAS was positively correlated with the proportion of hard choice in the 88% probability with large reward condition (*p* = 0.012), the proportion of hard choices in large reward condition (*p* = 0.048) and probability/reward difference score (*p* = 0.040), though all marginally failed to survive after Benjamini–Hochberg correction (Supplementary Table S1). Several significant correlations were observed between EEfRT performance indices and several cognitive test scores in schizotypy group (Supplementary Table S2) and in the entire sample (Supplementary Table S3), but none survived Bonferroni correction for multiple comparisons.

**Table 3:** Correlations between selected EEfRT performance indices with symptoms and functioning in schizotypy group^a^.

Variables	88% Probability		Large reward		88% Probability with large reward		Probability difference score		Reward difference score		Probability/Reward difference score	
	*r*_s_	*p*	*r*_s_	*p*	*r*_s_	*p*	*r*_s_	*p*	*r*_s_	*p*	*r*_s_	*p*
BNSS total	−0.120	0.459	−0.220	0.172	−0.274	0.087	−0.164	0.312	−0.308	0.053	−0.267	0.095
BNSS-AMO	−0.120	0.460	−0.134	0.410	−0.229	0.155	−0.256	0.110	−0.298	0.062	−0.289	0.070
C-SHAPS	−0.037	0.819	0.102	0.531	0.087	0.595	−0.053	0.747	0.134	0.409	0.048	0.769
SOFAS	−0.001	0.994	−0.129	0.428	0.189	0.243	0.245	0.128	0.119	0.463	0.303	0.058

AMO, Amotivation; BNSS, Brief Negative Symptom Scale; EEfRT, Effort Expenditure for Reward Task; C-SHAPS, Snaith-Hamilton Pleasure. Scale-Chinese version; SOFAS, Social and Occupational Functioning Assessment Scale. ^a^Spearman-rank correlation analyses were conducted.

## Discussion

The current study aimed to investigate effort-based decision-making and its relationship with amotivation and psychosocial functioning in individuals with schizotypy, using a physical effort allocation paradigm. Our analyses revealed three major findings. First, individuals with schizotypy (i.e., high-schizotypy) demonstrated comparable effort allocation performance with healthy controls (i.e., low-schizotypy). Second, schizotypy individuals with high levels of amotivation exhibited subtle alteration in effort allocation relative to schizotypy individuals with low levels of amotivation and controls. Correlation analyses also suggested that higher amotivational levels were negatively associated with worse effort-based decision-making performance. Third, our results suggested that diminished effort exertion was related to poorer psychosocial functioning.

To our knowledge, this is among the few studies to examine effort allocation in schizotypy. Our results were consistent with a previous study which failed to demonstrate significant difference between schizotypy individuals and controls in effort allocation for reward (36). This is also largely in line with the findings of another study which specifically examined effort-based decision-making, using the EEfRT task, among individuals with elevated positive schizotypy who were categorized according to the scores on the Perceptual Aberration and Magical Ideation scales (37). This study showed no main effect of group on effort allocation, though further analyses found that positive schizotypy group exerted less effortful choices than controls when the probability of reward was the lowest and the reward magnitude was the smallest (37). Of note, difference in defining schizotypy precludes direct comparison between this study and the current report. Taken together, contrary to our hypothesis and the respective literature on schizophrenia (20), the existing data (including our study results) generally indicated no reduced willingness of individuals with schizotypy to expend effort for high-value/high-probability reward relative to controls. Further research is also needed to clarify any potential differential relationships of effort-based decision-making with positive and negative schizotypy (53).

We adopted both categorical and dimensional approaches in quantifying severity of amotivation (52) in an attempt to comprehensively evaluate its relationship with effort allocation in schizotypy. We first subdivided individuals with schizotypy into those with high versus low levels of amotivation for comparison. Although our ANOVA revealed no group difference, we observed subtle abnormalities in effort-based decision-making in schizotypy individuals with high-amotivation relative to the counterparts with low-amotivation and healthy controls, with the former group showing significantly smaller difference in effort expenditure between small and large reward conditions as well as between low-probability/small-reward and high-probability/large-reward conditions than the latter two groups. Correlation analyses also revealed consistent findings with trend-level significance in the associations between amotivation levels and several EEfRT performance indices (i.e., reward difference score and probability/reward difference score) in schizotypy group. Hence, our results are generally in keeping with several previous studies on schizophrenia and first-episode psychosis, albeit in a subtler magnitude, which found that decreased willingness to expend effort for high-value/high-probability reward was most pronounced in patients with high levels of amotivation (21, 22, 26, 28). Conversely, one earlier study found that effort task performance was not related to self-reported state motivation in schizotypy individuals (36). Another study indicated that individuals with elevated social anhedonia, classified according to the scores on the Social Anhedonia Scale-Brief, displayed inefficient effort allocation rather than generalized or selected reduced effort expenditure (i.e., for high-value/high-probability reward), and made more effortful choices for reward than controls in trials with the lowest probability of reward and small reward magnitude, as well as under the condition of the greatest uncertainty for reward (i.e., 50% probability) in EEfRT (37). However, such discrepant findings might be attributable to cross-study variations in the choice of assessments for amotivation/negative symptoms (self-rated motivation scale was applied in McGovern (36); observer-rating scale BNSS was used in the current study; “social anhedonia” rather than a broader construct of amotivation was examined in McCarthy et al. (37)), and methods to classify individuals with (high) schizotypy [median split on the schizotypy assessment score in McGovern (36); extreme-group design based on SPQ-B scores in the current study; and “social anhedonia” rather than “schizotypy” construct studied in McCarthy et al. (37)]. Alternatively, emerging evidence has suggested that an experience-sampling method (ESM) involving multiple momentary symptom assessments in daily life might represent a more sensitive measurement of amotivation than conventional clinician- and self-rated symptom scales. In fact, recent data have demonstrated that amotivation assessed *via* ESM but not by clinician-administered symptom scale was correlated with EEfRT performance in schizophrenia patients (54, 55). Hence, future studies on effort allocation should consider to apply ESM for more refined assessment of amotivation in schizotypy individuals. In addition, recent research has proposed the need to take into consideration participants’ perception of task difficulty and effort in evaluating effort-based decision-making, and revealed that schizophrenia patients might be able to mobilize more effort to maintain performance despite their higher perceived task demands relative to controls (56). Further research is required to explore the relationships between effort allocation, perceived difficulty and effort, and amotivation in schizotypy individuals.

Our results that greater increase in effortful options from low-probability/small-reward to high-probability/large-reward conditions (i.e., probability/reward difference score) was marginally significantly correlated with better psychosocial functioning in schizotypy individuals accord with the findings of prior studies on effort task which showed that suboptimal effort allocation was related to greater functional impairment in schizophrenia patients (21, 24, 27, 33). Our additional analyses on the entire study sample even noted significant associations of SOFAS scores with several EEfRT performance indices, albeit marginally failed to survive after Benjamini–Hochberg correction. This thus provides supportive evidence suggesting the link between effort-based decision-making and real-world functional outcome in schizotypy individuals. There is some evidence indicating that cognitive deficits might be related to impaired effort-based decision-making in schizophrenia (23, 28, 32, 33), with worse cognitive performance being associated with decreased willingness to exert effort for reward. Overall, our analysis revealed a few significant correlations between cognitive variables and effort task performance indices in schizotypy group (and a few more in the entire sample), but none survived correction for multiple comparisons. Our findings may therefore suggest that cognitive functions might unlikely be specifically related to effort allocation performance in schizotypy individuals. Owing to the scarcity of existing data, more studies are needed to verify our findings on the relationships of effort-based decision-making with psychosocial functioning and cognitive performance in schizotypy.

Several limitations should be acknowledged in the current study. First, as longer duration is required to complete hard trials relative to easy trials, effort allocation performance might be confounded by temporal discounting (38). Second, we had modest sample size. The limited number of participants in each amotivation subgroup for comparison might hinder us from detecting other subtle yet significant differences. Third, our sample consisted of young people with narrow age range, and the study findings might therefore not be generalizable to people age older age. Fourth, although SPQ-B is a widely used and well-validated measurement for schizotypy, its relative brevity may render it less optimal to adequately capture schizotypical personality traits. Adoption of a more comprehensive assessment enabling delineation of various discrete schizotypy dimensions [e.g., (57)] may facilitate clarification of subtle association between schizotypy and deviated effect-allocation performance. Fifth, we did not have a measure of depressive symptoms, which have been found to be related to impaired effort-based decision-making (20, 58) and may confound the results on the association between amotivation and effort allocation. Sixth, it is acknowledged that effort-based decision-making can be classified on the basis of effort modalities, i.e., physical and cognitive effort, and accumulating data have indicated that these two effort modalities are mediated by related but distinct neural systems (59, 60). Until now, no study has been conducted to specifically examine cognitive effort-based decision-making in schizotypy individuals, and further investigation is warranted to clarify whether there are potential differential relationships between schizotypy and cognitive and physical allocation performance.

## Data availability statement

The raw data supporting the conclusions of this article will be made available by the authors, without undue reservation.

## Ethics statement

The studies involving human participants were reviewed and approved by Institutional Review Board of the University of Hong Kong/Hospital Authority Hong Kong West Cluster (HKU/HA HKW IRB). Written informed consent to participate in this study was provided by the participants’ legal guardian/next of kin.

## Author contributions

WC and CW designed and conceptualized the study. RC conducted statistical analysis and wrote the first draft of the manuscript. WC, CW, and CT interpreted the study data. WC revised and finalized the manuscript. All authors provided critical feedback to the manuscript and have approved the final manuscript.

## Funding

The study was supported by the Health and Medical Research Fund (Ref No.: 07181776), Food & Health Bureau of the HKSAR government.

## Conflict of interest

The authors declare that the research was conducted in the absence of any commercial or financial relationships that could be construed as a potential conflict of interest.

## Publisher’s note

All claims expressed in this article are solely those of the authors and do not necessarily represent those of their affiliated organizations, or those of the publisher, the editors and the reviewers. Any product that may be evaluated in this article, or claim that may be made by its manufacturer, is not guaranteed or endorsed by the publisher.

## References

[1] Rado S. Dynamics and classification of disordered behavior. Am J Psychiatry. (1953) 110:406–16. doi: 10.1176/ajp.110.6.406, PMID: 13104683

[2] Meehl PE. Toward an integrated theory of schizotaxia, schizotypy, and schizophrenia. J Pers Disord. (1990) 4:1–99. doi: 10.1521/pedi.1990.4.1.1

[3] Lenzenweger MF. Schizotypy: an organizing framework for schizophrenia research. Curr Dir Psychol Sci. (2006) 15:162–6. doi: 10.1111/j.1467-8721.2006.00428.x, PMID: 29945071

[4] Claridge G Beech T. Fully and quasi-dimensional constructions of schizotypy In: Raine A Lencz T, editors. Schizotypal Personality Disorder. Cambridge: Cambridge University Press (1995). 192–216.

[5] Kwapil TR Barrantes-Vidal N. Schizotypy: Looking back and moving forward. Schizophr Bull. (2015) 41:S366–73. doi: 10.1093/schbul/sbu186, PMID: 25548387 PMC4373633

[6] Nelson MT Seal ML Pantelis C Phillips LJ. Evidence of a dimensional relationship between schizotypy and schizophrenia: A systematic review. Neurosci Biobehav Rev. (2013) 37:317–27. doi: 10.1016/j.neubiorev.2013.01.004, PMID: 23313650

[7] Ettinger U Meyhöfer I Steffens M Wagner M Koutsouleris N. Genetics, cognition, and neurobiology of schizotypal personality: A review of the overlap with schizophrenia. Front Psych. (2014) 5:1–16. doi: 10.3389/fpsyt.2014.00018 PMC3931123 24600411

[8] Siddi S Petretto DR Preti A. Neuropsychological correlates of schizotypy: a systematic review and meta-analysis of cross-sectional studies. Cogn Neuropsychiatry. (2017) 22:186–212. doi: 10.1080/13546805.2017.1299702, PMID: 28288547

[9] Kirschner M Hodzic-Santor B Antoniades M Nenadic I Kircher T Krug A. Cortical and subcortical neuroanatomical signatures of schizotypy in 3004 individuals assessed in a worldwide ENIGMA study. Mol Psychiatry. (2022) 27:1167–76. doi: 10.1038/s41380-021-01359-9, PMID: 34707236 PMC9054674

[10] Barrantes-Vidal N Grant P Kwapil TR. The role of schizotypy in the study of the etiology of schizophrenia spectrum disorders. Schizophr Bull. (2015) 41:S408–16. doi: 10.1093/schbul/sbu191, PMID: 25810055 PMC4373635

[11] Messinger JW Trémeau F Antonius D Mendelsohn E Prudent V Stanford AD. Avolition and expressive deficits capture negative symptom phenomenology: implications for DSM-5 and schizophrenia research. Clin Psychol Rev. (2011) 31:161–8. doi: 10.1016/j.cpr.2010.09.002, PMID: 20889248 PMC2997909

[12] Strauss GP Bartolomeo LA Harvey PD. Avolition as the core negative symptom in schizophrenia: relevance to pharmacological treatment development. NPJ Schizophr. (2021) 7:16. doi: 10.1038/s41537-021-00145-4, PMID: 33637748 PMC7910596

[13] Fervaha G Foussias G Agid O Remington G. Motivational deficits in early schizophrenia: Prevalent, persistent, and key determinants of functional outcome. Schizophr Res. (2015) 166:9–16. doi: 10.1016/j.schres.2015.04.040, PMID: 25982811

[14] Chang WC Hui CLM Chan SKW Lee EHM Chen EYH. Impact of avolition and cognitive impairment on functional outcome in first-episode schizophrenia-spectrum disorder: A prospective one-year follow-up study. Schizophr Res. (2016) 170:318–21. doi: 10.1016/j.schres.2016.01.004, PMID: 26778673

[15] Chang WC Kwong VWY Or Chi Fai P Lau ESK Chan GHK Jim OTT. Motivational impairment predicts functional remission in first-episode psychosis: 3-Year follow-up of the randomized controlled trial on extended early intervention. Aust N Z J Psychiatry. (2018) 52:1194–201. doi: 10.1177/0004867418758918, PMID: 29475381

[16] Strauss GP Waltz JA Gold JM. A review of reward processing and motivational impairment in schizophrenia. Schizophr Bull. (2014) 40:107–16. doi: 10.1093/schbul/sbt197 PMC3934394 24375459

[17] Waltz JA Gold JM. Motivational deficits in schizophrenia and the representation of expected value. Curr Top Behav Neurosci. (2016) 27:375–410. doi: 10.1007/7854_2015_385, PMID: 26370946 PMC4792780

[18] Green MF Horan WP Barch DM Gold JM. Effort-based decision making: A novel approach for assessing motivation in schizophrenia. Schizophr Bull. (2015) 41:1035–44. doi: 10.1093/schbul/sbv071, PMID: 26089350 PMC4535644

[19] Gold JM Waltz JA Frank MJ. Effort cost computation in schizophrenia: a cmmentary on the recent literature. Biol Psychiatry. (2015) 78:747–53. doi: 10.1016/j.biopsych.2015.05.005, PMID: 26049208 PMC4636936

[20] Culbreth AJ Moran EK Barch DM. Effort-cost decision-making in psychosis and depression: Could a similar behavioral deficit arise from disparate psychological and neural mechanisms? Psychol Med. (2018) 48:889–904. doi: 10.1017/S0033291717002525, PMID: 28889803

[21] Chang WC Chu AOK Treadway MT Strauss GP Chan SKW Lee EHM. Effort-based decision-making impairment in patients with clinically-stabilized first-episode psychosis and its relationship with amotivation and psychosocial functioning. Eur Neuropsychopharmacol. (2019) 29:629–42. doi: 10.1016/j.euroneuro.2019.03.006, PMID: 30879927

[22] Chang WC Westbrook A Strauss GP Chu AOK Chong CSY Siu CMW. Abnormal cognitive effort allocation and its association with amotivation in first-episode psychosis. Psychol Med. (2020) 50:2599–609. doi: 10.1017/S0033291719002769, PMID: 31576787

[23] Serper M Payne E Dill C Portillo C Taliercio J. Allocating effort and anticipating pleasure in schizophrenia: Relationship with real world functioning. Eur Psychiatry. (2017) 46:57–64. doi: 10.1016/j.eurpsy.2017.07.008, PMID: 29031122

[24] Strauss GP Whearty KM Morra LF Sullivan SK Ossenfort KL Frost KH. Avolition in schizophrenia is associated with reduced willingness to expend effort for reward on a progressive ratio task. Schizophr Res. (2016) 170:198–204. doi: 10.1016/j.schres.2015.12.006, PMID: 26701649 PMC4707087

[25] Hartmann MN Hager OM Reimann AV Chumbley JR Kirschner M Seifritz E. Apathy but not diminished expression in schizophrenia is associated with discounting of monetary rewards by physical effort. Schizophr Bull. (2015) 41:503–12. doi: 10.1093/schbul/sbu102, PMID: 25053653 PMC4332944

[26] Fervaha G Duncan M Foussias G Agid O Faulkner GE Remington G. Effort-based decision making as an objective paradigm for the assessment of motivational deficits in schizophrenia. Schizophr Res. (2015) 168:483–90. doi: 10.1016/j.schres.2015.07.023, PMID: 26215506

[27] Barch DM Treadway MT Schoen N. Effort, anhedonia, and function in schizophrenia: reduced effort allocation predicts amotivation and functional impairment. J Abnorm Psychol. (2014) 123:387–97. doi: 10.1037/a0036299, PMID: 24886012 PMC4048870

[28] Gold JM Strauss GP Waltz JA Robinson BM Brown JK Frank MJ. Negative symptoms of schizophrenia are associated with abnormal effort-cost computations. Biol Psychiatry. (2013) 74:130–6. doi: 10.1016/j.biopsych.2012.12.022, PMID: 23394903 PMC3703817

[29] Docx L de La Asuncion J Sabbe B Hoste L Baeten R Warnaerts N. Effort discounting and its association with negative symptoms in schizophrenia. Cogn Neuropsychiatry. (2015) 20:172–85. doi: 10.1080/13546805.2014.993463, PMID: 25559619

[30] Treadway MT Peterman JS Zald DH Park S. Impaired effort allocation in patients with schizophrenia. Schizophr Res. (2015) 161:382–5. doi: 10.1016/j.schres.2014.11.024, PMID: 25487699 PMC4308548

[31] McCarthy JM Treadway MT Bennett ME Blanchard JJ. Inefficient effort allocation and negative symptoms in individuals with schizophrenia. Schizophr Res. (2016) 170:278–84. doi: 10.1016/j.schres.2015.12.017, PMID: 26763628 PMC4740196

[32] Bismark AW Thomas ML Tarasenko M Shiluk AL Rackelmann SY Young JW. Relationship between effortful motivation and neurocognition in schizophrenia. Schizophr Res. (2018) 193:69–76. doi: 10.1016/j.schres.2017.06.042, PMID: 28673753 PMC5754266

[33] Horan WP Felice Reddy L Barch DM Buchanan RW Dunayevich E Gold JM. Effort-based decision-making paradigms for clinical trials in schizophrenia: Part 2 - external validity and correlates. Schizophr Bull. (2015) 41:1055–65. doi: 10.1093/schbul/sbv090, PMID: 26209546 PMC4535650

[34] Cohen AS Davis TE. Quality of life across the schizotypy spectrum: findings from a large nonclinical adult sample. Compr Psychiatry. (2009) 50:408–14. doi: 10.1016/j.comppsych.2008.11.002, PMID: 19683610

[35] Kwapil TR Mielock A Chun CA Kemp KC Sperry SH Gross GM. Association of multidimensional schizotypy with psychotic-like experiences, affect, and social functioning in daily life: Comparable findings across samples and schizotypy measures. J Abnorm Psychol. (2020) 129:492–504. doi: 10.1037/abn0000522, PMID: 32250141

[36] Mcgovern JE. (2014). *Effort-based decision-making in schizotypy*. Master Thesis, Louisiana: Louisiana State University.

[37] McCarthy JM Treadway MT Blanchard JJ. Motivation and effort in individuals with social anhedonia. Schizophr Res. (2015) 165:70–5. doi: 10.1016/j.schres.2015.03.030, PMID: 25888337 PMC4437913

[38] Treadway MT Buckholtz JW Schwartzman AN Lambert WE Zald DH. Worth the “EEfRT”? The effort expenditure for rewards task as an objective measure of motivation and anhedonia. PLoS One. (2009) 4:e6598. doi: 10.1371/journal.pone.0006598 19672310 PMC2720457

[39] Treadway MT Buckholtz JW Cowan RL Woodward ND Li R Ansari MS. Dopaminergic mechanisms of individual differences in human effort-based decision-making. J Neurosci. (2012) 32:6170–6. doi: 10.1523/JNEUROSCI.6459-11.2012, PMID: 22553023 PMC3391699

[40] Reddy LF Horan WP Barch DM Buchanan RW Dunayevich E Gold JM. Effort-based decision-making paradigms for clinical trials in schizophrenia: Part 1 - psychometric characteristics of 5 paradigms. Schizophr Bull. (2015) 41:1045–54. doi: 10.1093/schbul/sbv089, PMID: 26142081 PMC4535649

[41] Wong CS Hui CL Suen YN Wong SM Chang WC Chan SK. The Hong Kong Youth Epidemiological Study of Mental Health (HK-YES): a population-based psychiatric epidemiology study of youth mental health in Hong Kong: a study protocol. Early Interv Psychiatry. (2023). doi: 10.1111/eip.13364 Online ahead of print, PMID: 36632706

[42] Raine A Benishay D. The SPQ-B: A brief screening instrument for schizotypal personality disorder. J Personal Disord. (1995) 9:346–55. doi: 10.1521/pedi.1995.9.4.346, PMID: 32091308

[43] Ma WF Wu PL Yang SJ Cheng KF Chiu HT Lane HY. Sensitivity and specificity of the Chinese version of the Schizotypal Personality Questionnaire-Brief for identifying undergraduate students susceptible to psychosis. Int J Nurs Stud. (2010) 47:1535–44. doi: 10.1016/j.ijnurstu.2010.05.010, PMID: 20580002

[44] Kirkpatrick B Strauss GP Nguyen L Fischer BA Daniel DG Cienfuegos A. The brief negative symptom scale: Psychometric properties. Schizophr Bull. (2011) 37:300–5. doi: 10.1093/schbul/sbq059, PMID: 20558531 PMC3044634

[45] Strauss GP Hong LE Gold JM Buchanan RW McMahon RP Keller WR. Factor structure of the brief negative symptom scale. Schizophr Res. (2012) 142:96–8. doi: 10.1016/j.schres.2012.09.007, PMID: 23062750 PMC3502636

[46] Liu WH Wang LZ Zhu YH Li MH Chan RCK. Clinical utility of the Snaith-Hamilton-Pleasure scale in the Chinese settings. BMC Psychiatry. (2012) 12:2–7. doi: 10.1186/1471-244X-12-184 23110667 PMC3549923

[47] Goldman HH Skodol AE Lave TR. Revising axis V for DSM-IV: a review of measures of social functioning. Am J Psychiatry. (1992) 149:1148–56. doi: 10.1176/ajp.149.9.1148, PMID: 1386964

[48] Hong Kong Psychological Society. The Wechsler Adult Memory Scale-Revised (Cantonese version). Hong Kong: Hong Kong Psychological Society (1989).

[49] Hong Kong Psychological Society. The Wechsler Adult Intelligence Scale-Revised (Cantonese version). Hong Kong: Hong Kong Psychological Society (1989).

[50] Reitan RM. The relation of the Trail Making Test to organic brain damage. J Consult Psychol. (1955) 19:393–4. doi: 10.1037/h0044509, PMID: 13263471

[51] Diller L Ben-Yishay Y Gerstman LJ Goodin R Gordon W Weinberg J. Studies in Scanning Behavior in Hemiplegia. Rehabilitation Monograph No. 50, Studies in Cognition and Rehabilitation in Hemiplegia. New York, NY: New York University Medical Center, Institute of Rehabilitation Medicine (1974).

[52] Ahmed AO Strauss GP Buchanan RW Kirkpatrick B Carpenter WT. Are negative symptoms dimensional or categorical detection and validation of deficit schizophrenia with taxometric and latent variable mixture models. Schizophr Bull. (2015) 41:879–91. doi: 10.1093/schbul/sbu163, PMID: 25399026 PMC4466177

[53] Yan Y Hu H Wang L Zhang Y Lui SSY Huang J. Negative schizotypal traits predict the reduction of reward motivation in effort – reward imbalance. Eur Arch Psychiatry Clin Neurosci. (2022) Online ahead of print). doi: 10.1007/s00406-022-01419-3, PMID: 35637380

[54] Gard DE Sanchez AH Cooper K Fisher M Garrett C Vinogradov S. Do people with schizophrenia have difficulty anticipating pleasure, engaging in effortful behavior, or both? J Abnorm Psychol. (2014) 123:771–82. doi: 10.1037/abn0000005, PMID: 25133986 PMC4227944

[55] Moran EK Culbreth AJ Barch DM. Ecological momentary assessment of negative symptoms in schizophrenia: Relationships to effort-based decision making and reinforcement learning. J Abnorm Psychol. (2017) 126:96–105. doi: 10.1037/abn0000240, PMID: 27893230 PMC5433621

[56] Décombe A Brunel L Capdevielle D Raffard S. Too much or too little? Exploring effort perception in schizophrenia within the framework of motivational intensity theory. Cogn Neuropsychiatry. (2020) 25:312–27. doi: 10.1080/13546805.2020.1798220, PMID: 32727294

[57] Kwapil TR Gross GM Silvia PJ Raulin ML Barrantes-Vidal N. Development and psychometric properties of the Multidimensional Schizotypy Scale: A new measure for assessing positive, negative, and disorganized schizotypy. Schizophr Res. (2018) 193:209–17. doi: 10.1016/j.schres.2017.07.001, PMID: 28735642

[58] Treadway MT Bossaller NA Shelton RC Zald DH. Effort-based decision-making in major depressive disorder: a translational model of motivational anhedonia. J Abnorm Psychol. (2012) 121:553–8. doi: 10.1037/a0028813, PMID: 22775583 PMC3730492

[59] Schmidt L Lebreton M Cléry-Melin ML Daunizeau J Pessiglione M. Neural mechanisms underlying motivation of mental versus physical effort. PLoS Biol. (2012) 10:e1001266. doi: 10.1371/journal.pbio.1001266, PMID: 22363208 PMC3283550

[60] Chong TTJ Apps M Giehl K Sillence A Grima LL Husain M. Neurocomputational mechanisms underlying subjective valuation of effort costs. PLoS Biol. (2017) 15:e1002598. doi: 10.1371/journal.pbio.1002598, PMID: 28234892 PMC5325181

