# Supplementary material for: Effort-based decision making in schizotypy and its relationship with amotivation and psychosocial functioning
Source: Front Psychiatry. 2023 Feb 16;14:1123046. doi: 10.3389/fpsyt.2023.1123046 (PMC9978481; doi:10.3389/fpsyt.2023.1123046)
Supplement: Supplementary file 1 [file Table_1.DOCX]

Supplementary Material

1. **Supplementary Table 1**. Correlations between selected EEfRT performance indices with symptoms severity in the whole sample.
2. **Supplementary Table 2**. Correlations between selected EEfRT performance indices and cognitive performances in schizotypy group.
3. **Supplementary Table 3**. Correlations between selected EEfRT performance indices and cognitive performances in the whole sample.

**Supplementary Table 1** Correlations between selected EEfRT performance indices with symptoms severity in the whole study sample.^a^

| Variables | 88% Probability | | Large Reward | | 88% Probability with Large Reward | | Probability Difference Score | | Reward Difference Score | | Probability/Reward Difference Score | | |
| --- | --- | --- | --- | --- | --- | --- | --- | --- | --- | --- | --- | --- | --- |
|  | r_s_ | *p* | r_s_ | *p* | r_s_ | *p* | r_s_ | *p* | r_s_ | *p* | r_s_ | *p* |  |
| BNSS total | -0.140 | 0.216 | -0.195 | 0.083 | -0.245 | 0.029^c^ | -0.054 | 0.633 | -0.170 | 0.132 | -0.105 | 0.353 |  |
| BNSS-AMO | -0.092 | 0.419 | -0.119 | 0.293 | -0.160 | 0.155 | -0.072 | 0.524 | -0.136 | 0.229 | -0.069 | 0.546 |  |
| C-SHAPS | 0.038 | 0.741 | 0.040 | 0.721 | 0.027 | 0.810 | -0.007 | 0.950 | 0.046 | 0.682 | 0.067 | 0.555 |  |
| SOFAS | 0.074 | 0.516 | 0.048^e^ | 0.673 | 0.279 | 0.012^b^ | 0.178 | 0.115 | 0.143 | 0.207 | 0.230 | 0.040 ^d^ |  |

AMO, Amotivation; BNSS, Brief Negative Symptom Scale; C-SHAPS, Snaith-Hamilton Pleasure Scale-Chinese version; EEfRT, Effort Expenditure for Reward Task; SOFAS, Social and Occupational Functioning Assessment Scale.

^a^ Spearman-rank correlation analyses were conducted.

^b^ Benjamini–Hochberg procedure indicated that the significant correlation (*p* value of 0.012 > critical value of 0.010) marginally failed to survive correction for multiple comparisons.

^c^ Benjamini–Hochberg procedure indicated that the significant correlation (*p* value of 0.029 > critical value of 0.021) marginally failed to survive correction for multiple comparisons.

^d^ Benjamini–Hochberg procedure indicated that the significant correlation (*p* value of 0.040 > critical value of 0.031) marginally failed to survive correction for multiple comparisons.

^e^ Benjamini–Hochberg procedure indicated that the significant correlation (*p* value of 0.048 > critical value of 0.042) marginally failed to survive correction for multiple comparisons.

**Supplementary Table 2** Correlations between selected EEfRT performance indices and cognitive performances in schizotypy group.^a^

| Variables^b^ | 88% Probability | | Large Reward | | 88% Probability with Large Reward | | Probability Difference Score | | Reward Difference Score | | Probability/Reward Difference Score | |
| --- | --- | --- | --- | --- | --- | --- | --- | --- | --- | --- | --- | --- |
|  | r_s_ | *p* | r_s_ | *p* | r_s_ | *p* | r_s_ | *p* | r_s_ | *p* | r_s_ | *p* |
| Logical memory | 0.003 | 0.987 | -0.041 | 0.811 | 0.152 | 0.375 | 0.141 | 0.413 | -0.005 | 0.978 | 0.161 | 0.347 |
| Digit symbol | 0.055 | 0.736 | 0.101 | 0.534 | 0.274 | 0.087 | 0.144 | 0.374 | 0.142 | 0.382 | 0.215 | 0.184 |
| Letter number span | 0.122 | 0.452 | -0.105 | 0.521 | 0.148 | 0.361 | 0.329 | 0.038^c^ | 0.027 | 0.869 | 0.175 | 0.281 |
| Trail making A | 0.151 | 0.351 | 0.259 | 0.107 | -0.033 | 0.838 | -0.209 | 0.196 | 0.058 | 0.723 | -0.120 | 0.461 |
| Trail making B | -0.068 | 0.676 | -0.010 | 0.951 | -0.352 | 0.026^c^ | -0.247 | 0.124 | -0.053 | 0.744 | -0.284 | 0.076 |
| Letter cancellation | -0.145 | 0.371 | -0.075 | 0.647 | 0.109 | 0.502 | -0.022 | 0.891 | 0.078 | 0.632 | 0.197 | 0.223 |

EEfRT, Effort Expenditure for Reward Task.

^a^ Spearman-rank correlation analyses were conducted.

^b^ Bonferroni correction was applied to all the correlations (critical value=0.00119).

^c^ Failed to survive Bonferroni correction for multiple comparisons.

**Supplementary Table 3** Correlations between selected EEfRT performance indices and cognitive performances in the whole study sample.^a^

| Variables^b^ | 88% Probability | | Large Reward | | 88% Probability with Large Reward | | Probability Difference Score | | | Reward Difference Score | | Probability/Reward Difference Score | |
| --- | --- | --- | --- | --- | --- | --- | --- | --- | --- | --- | --- | --- | --- |
|  | r_s_ | *p* | r_s_ | *p* | r_s_ | *p* | | r_s_ | *p* | r_s_ | *p* | r_s_ | *p* |
| Logical memory | -0.025 | 0.833 | -0.050 | 0.667 | 0.098 | 0.404 | | 0.083 | 0.480 | 0.001 | 0.993 | 0.144 | 0.217 |
| Digit symbol | -0.016 | 0.888 | 0.028 | 0.804 | 0.121 | 0.284 | | 0.065 | 0.566 | 0.101 | 0.371 | 0.116 | 0.305 |
| Letter number span | 0.190 | 0.092 | 0.059 | 0.604 | 0.242 | 0.030^c^ | | 0.298 | 0.007^c^ | 0.154 | 0.174 | 0.289 | 0.009^c^ |
| Trail making A | 0.177 | 0.115 | 0.178 | 0.114 | 0.063 | 0.579 | | 0.012 | 0.917 | 0.091 | 0.422 | 0.061 | 0.591 |
| Trail making B | 0.015 | 0.897 | -0.004 | 0.971 | -0.228 | 0.042^c^ | | -0.127 | 0.263 | -0.044 | 0.701 | -0.166 | 0.141 |
| Letter cancellation | -0.041 | 0.717 | -0.033 | 0.769 | 0.212 | 0.059 | | 0.109 | 0.336 | 0.111 | 0.326 | 0.292 | 0.009^c^ |

EEfRT, Effort Expenditure for Reward Task.

^a^ Spearman-rank correlation analyses were conducted.

^b^ Bonferroni correction was applied to all the correlations (critical value=0.00119).

^c^ Failed to survive Bonferroni correction for multiple comparisons.
